# A Comparative Study of Total Knee Arthroplasty and Unicondylar Knee Arthroplasty in the Treatment of Knee Osteoarthritis

**DOI:** 10.1155/2022/7795801

**Published:** 2022-04-28

**Authors:** Lin Wang, Qiang Wang, Qiuwei Li, Fahuan Song

**Affiliations:** ^1^Department of Orthopedics, Yijishan Hospital, Wannan Medical College, Wuhu 241000, Anhui, China; ^2^Department of Nuclear Medicine, Zhejiang Provincial People's Hospital, Affiliated People's Hospital, Hangzhou Medical College, Hangzhou 310014, Zhejiang, China; ^3^Key Laboratory of Endocrine Gland Diseases of Zhejiang Province, Hangzhou, Zhejiang, China

## Abstract

**Objective:**

To compare the clinical efficacy of total knee arthroplasty (TKA) and unicondylar knee arthroplasty (UKA) in the treatment of knee osteoarthritis (KOA).

**Methods:**

A retrospective analysis was conducted on 30 patients admitted to the Department of Orthopaedics of Yijishan Hospital from 2020 to 2021. The patients were divided into UKA group (*n* = 15) and TKA group (control, *n* = 15). The intraoperative situation and postoperative clinical indicators of patients in the two groups were collected and compared, such as operation time, intraoperative blood loss, length of hospital stay, postoperative complications, and postoperative functional recovery. Postoperative functional recovery was investigated by the visual analogue pain scale (VAS), knee score scale (HSS), and knee range of motion (ROM) scores 5 days after surgery.

**Results:**

Perioperative indexes in the UKA group were significantly lower than those in the TKA group, including operation time, intraoperative blood loss, first time going to the ground, and length of hospital stay. VAS, HSS, and ROM scores in the two groups were significantly improved after surgery compared with those before surgery. However, ROM scores in the UKA group were significantly better than in the TKA group. In terms of early postoperative complications, there was one case of venous thrombosis of lower limbs in the UKA group, while in the TKA group there was one case of delayed wound healing due to diabetes, and one case of deep infection.

**Conclusion:**

Both UKA and TKA are very successful options for the treatment of KOA, but the use of UKA can promote the recovery of postoperative knee function, reduce postoperative complications, and achieve more satisfactory than expected results.

## 1. Introduction

Knee osteoarthritis (KOA) is considered to be one of the most common musculoskeletal diseases, which can lead to joint degeneration, resulting in impairment of activities of daily living [1]. It is characterized by pain, cartilage loss, and joint inflammation [2]. Osteoarthritis is generally a slowly progressive disorder. At least 1 in 7 people with incident knee osteoarthritis develop an abrupt progression to advanced-stage radiographic disease, many within 12 months [3].

The treatment methods are mainly divided into nonsurgical treatment and surgical treatment. Nonoperative treatment includes patient education, lifestyle changes, and the use of orthopaedic devices. These can be achieved in the community. Surgical options include joint preservation surgery, such as arthroscopic osteotomy or joint replacement. Joint replacement surgery can be performed separately, such as patellofemoral joint replacement, unicondylar knee arthroplasty (UKA), and total knee arthroplasty (TKA) [4]. TKA makes patients highly satisfied because it provides patients with considerable medium- and long-term benefits in terms of quality of life, pain relief, and function [5]. Although total knee arthroplasty is an effective treatment for knee arthritis, according to relevant literature, up to 30% of patients are dissatisfied [6]. The reasons for these dissatisfactions come from many physical, behavioral, social, and psychological factors, as well as some postoperative complications such as thrombosis, infection and loosening or uneven arrangement of prosthetic parts [6, 7]. UKA is a bone-preserving and ligament-preserving procedure [8]. Studies have shown that this procedure restores intrinsic knee kinematics and that patient satisfaction is superior to TKA [9]. However, the durability of UKA and the need for revision surgery have been issues of concern [10, 11]. In order to explore the efficacy of TKA and UKA in patients with KOA, 30 patients with KOA treated in the Department of orthopaedics of Yijishan Hospital from November 2020 to July 2021 were included, and then the differences between the two groups in each observation index were compared.

## 2. Materials and Methods

### 2.1. General Information

30 patients with KOA treated in Yijishan Hospital of Wannan Medical College from 2020 to 2021 were included in this study and were divided into the UKA group and TKA group according to different operation methods. This study was approved by the Medical Ethics Committee of the Yijishan Hospital, Wannan Medical College.

### 2.2. Inclusion and Exclusion Criteria

The inclusion criteria were as follows: (a) patients with end-stage primary knee osteoarthritis; (b) symptomatic knee osteoarthritis; (c) patients who underwent TKA or UKA for the first time.

The exclusion criteria are as follows: (a) patients with basic diseases of important tissues and organs such as heart, liver, brain, and kidney; (b) patients with severe mental diseases and unable to cooperate; (c) patients with severe knee deformity.

### 2.3. UKA Surgery

**Figure 1:**
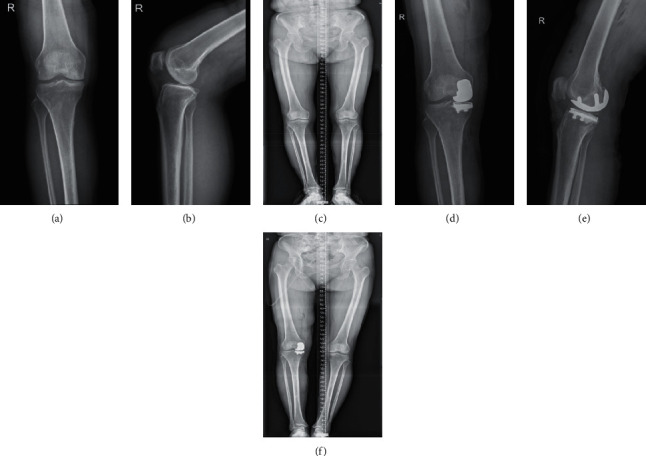
Patient, female, 53 years old. Pain and limited movement for more than 4 years, osteoarthritis of the right knee, unicondylar knee arthroplasty of the right knee. (a, b) Preoperative anteroposterior and lateral view of right knee joint. DR: the right knee joint was composed of complete bone, some bone edges showed hyperosteogeny, intercondylar protuberant hyperplasia, joint space existed, and there were no obvious abnormalities in surrounding soft tissues. Degeneration of the right knee. (c) Preoperative full-length X-ray of lower limbs showed narrowing of the medial joint space of the right knee joint and valgus deformity. (d, e) Anteroposterior and lateral radiographs of the right knee joint after partial replacement of the right knee joint: the replacement was in place and the joint space was called. (f) Postoperative full-length X-ray of lower limbs showed that the knee valgus deformity was corrected, the prosthesis was in a good position, and the force line of lower limbs was restored.

(1) First, the patient is placed in a horizontal position and under combined spinal and epidural anesthesia. An incision is made along the medial incision of the knee, so that a medial parapatellar capsule is dissected. The distal end of the medial femoral condyle was seen, and the osteophyte is removed. The tibial guide is installed, and the medial tibial plateau osteotomy is performed along the guide. (2) The distal end of the medial femoral condyle and the posterior condyle cartilage are removed with a swing saw. Then, a femoral condyle osteotomy module of appropriate size is selected, and the module is placed in the center of the medial femoral condyle, fixing it with two short fixing screws. (3) Holes are drilled along the guide, the edge of the femoral guide drill is marked, and all medial cartilage within the marked range was removed. The femoral single condyle prosthesis and the tibial flat platform prosthesis test model was installed and the force line was tested, the knee joint was straightened and bended. (4) Bone cement was used to fix the femoral prosthesis and tibial prosthesis, the polyethylene pad was inserted, and a drainage tube was placed. The parapatellar support belt and subcutaneous skin was sutured, and wrapped with sterile dressing. (5) The amount of intraoperative bleeding was recorded, the intraoperative anesthesia was satisfied, and returned to the ward after operation.  Figure 1 illustrates a patient treated with UKA of the right knee.

### 2.4. TKA Surgery

**Figure 2:**
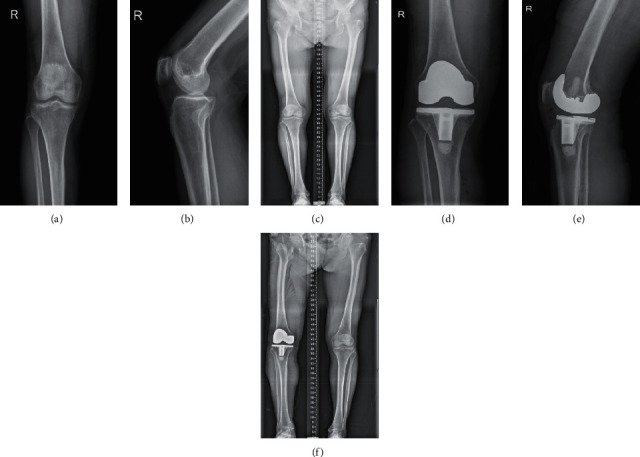
Patient, female, 67 years old. The pain in the right knee has been aggravated for more than 10 years. The osteoarthritis of the right knee joint was treated with total knee arthroplasty of the right knee. (a, b) The bone composition of the right knee joint is complete, the bone hyperplasia shadow can be seen at the bone edge, the intercondylar eminence hyperplasia, the joint space is under weighed, and there is no obvious abnormality in the surrounding soft tissue. Degeneration of the right knee. (c) Preoperative X-ray of the full length of the lower limb showed that the left knee was obviously valgus deformity and the patella was in the middle. (d, e) Replacement in place, joint space, etc. Changes after right knee arthroplasty. (f) The postoperative X-ray of the whole length of the lower limb showed that the valgus deformity of the knee joint was corrected, the prosthesis was in a good position, and the force line of the lower limb was restored.

(1) Patients were placed in a supine position with combined spinal and epidural anesthesia. An anterior median surgical incision was made, incise was made sharply until the patellar surface is free on both sides of the patellar surface, the medial patella to the medial patellar tendon was cut down along, the medial synovial tissue and meniscus were removed, and the medial acetabular retractor was placed in the goose foot bursa. (2) In the knee extension position, the attachment point of the meniscus was cut off in front of the lateral tibial platform to the outside of the platform, the patella was turned over and the knee was bended, the synovium was removed, the deep sliding capsule and fat at the lower part of the patellar tendon were removed, the retractor was pulled into the internal and external platform, the whole tibial platform was exposed, the front fork was cut off, and the meniscus was removed on both sides. The femoral and tibial marrow were reamed, respectively, and the osteophytes were cleaned up. (3) The tibial model was selected according to the test results. (4) The right prosthesis was selected, bone cement was applied to the posterior condyle of the femoral prosthesis, and bone cement was applied to the three sides before osteotomy. The femoral prosthesis was inserted according to the intramedullary opening and prosthesis fixing hole, the marginal bone cement was removed, and the tibial polyethylene gasket was inserted. (5) The drainage tube was placed, the parapatellar retinaculum and subcutaneous skin were sutured, the articular cavity was injected with tranexamic acid, and finally wrapped with sterile dressing. (6) The intraoperative bleeding was recorded, the intraoperative anesthesia was satisfactory, and the patients returned to the ward after the operation.  Figure 2 shows a patient treated with TKA.

### 2.5. Observation Indicators

Visual analogue pain scale (VAS), knee score scale (HSS), and knee range of motion (ROM) were used before operation and on the 5th day after the operation. At the same time, the perioperative clinical conditions of the two groups were recorded, including operation time, intraoperative bleeding, postoperative landing time, and hospital stay. The two groups were followed up for 12 months to carefully record the postoperative related complications, including delayed wound healing, superficial or deep infection, and lower extremity venous thrombosis.

### 2.6. Statistical Analysis

The numerical data were expressed as mean ± standard deviation (SD). All statistical analyses in this study were performed using the SPSS 22.0 software (IBM Corp., Armonk, NY, USA). The paired *t*-test was used for quantitative data of normal distribution. The qualitative data between the two groups were tested by *χ*^2^ test. *P* < 0.05 was considered significant.

## 3. Results

### 3.1. Patients Information

There were 4 males and 11 females in the UKA group. The age ranged from 53 to 79 years, with an average age of 64.9 years. In the TKA group, there were 4 males and 11 females. The age ranged from 57 to 83 years, with an average age of 69.8 years. There was no significant difference between the two groups in general baseline data such as age, gender, BMI, and operation side, which was comparable. The general preoperative data of the two groups were shown in Table 1.

### 3.2. The Perioperative Indexes of the Two Groups were Compared

As shown in Table 2, the operation time, postoperative walking time, and hospitalization time in the UKA group were significantly shorter than those of the TKA group, and the amount of intraoperative bleeding was also significantly reduced (*P* < 0.05).

### 3.3. Comparison of HSS Score and VAS Score before and after Operation

Further, there was no significant difference in preoperative VAS and HSS scores between the two groups (*P* > 0.05). However, the VAS and HSS scores of the two groups were significantly improved 5 days after the operation (*P* < 0.01) (Table 3).

### 3.4. Comparison of Knee ROM Changes before and after Operation

Next, we observed the postoperative range of motion in both groups. There was no significant difference in ROM scores between the two groups before operation and 5 days after the operation (*P* > 0.05). When compared with before operation, the ROM scores of the two groups were significantly improved 5 days after the operation (*P* < 0.01), and the ROM scores in the UKA group were significantly better than those of the TKA group (*P* < 0.01) (Table 4).

### 3.5. Comparison of Postoperative Complications between the Two Groups

Finally, the early postoperative complications of the two groups were compared. In the UKA group, there was 1 patient with lower limb venous thrombosis, while 1 patient in the TKA group had delayed wound healing due to diabetes and 1 patient had a deep infection. However, there was no significant difference in the total number of complications between the two groups (*P* > 0.05) (Table 5).

## 4. Discussion

KOA is a common and frequently occurring disease in the middle-aged and elderly. It is a degenerative disease whose prevalence increases significantly with age. At present, the treatment of KOA can be divided into nonsurgical treatment or surgical treatment. Nondrug treatment includes the core first-line treatment for all patients with osteoarthritis, including education, self-management, exercise and weight loss. At present, drug treatment mainly includes nonsteroidal anti-inflammatory drugs (NSAIDs) or joint endothelial steroids [12]. Surgical treatment is the most effective means of end-stage KOA. At present, the main types of surgical methods include patellofemoral arthroplasty, UKA, or TKA.

UKA is an alternative method to preserve bone and ligament in total knee arthroplasty for patients with end-stage univentricular degeneration of the knee joint [13]. In the past 30 years, the clinical application of single condylar arthroplasty has been fully developed [14]. Kozinn and Scott [15] creatively proposed the best indications of UKA in 1987, including osteoarthritis/osteonecrosis of single medial or lateral condyle of the knee joint, aged less than 60 years old, weight less than 82 kg, and flexion contracture angle less than 5°. However, with the continuous development of single condylar knee arthroplasty, people gradually realize that if only a few patients with knee osteoarthritis meet the conditions of UKA according to the standards [15], the indications of UKA are expanding. At present, the indications of UKA can be defined as anterior medial osteoarthritis with complete cruciate ligament and medial collateral ligament. The advantage of UKA is that the proposed advantages include shorter and faster recovery time, lower incidence, higher functional activity due to normal knee kinematics, and the subjective feeling of normal knee joint caused by the retention of anterior and posterior cruciate ligament and part of meniscus [16]. However, at present, the disadvantage is that UKA has a higher revision rate and a lower service life of the prosthesis. However, this may be due to the material of the prosthesis. With the continuous updating of surgeons' technology and the continuous improvement of prosthesis materials, the postoperative revision rate will be greatly reduced so that patients can benefit from lifelong results [8, 17]. TKA is a very mature surgical technology, especially for patients over 70 years old with advanced end-stage knee osteoarthritis. The long-term effect is very good, and can greatly improve their quality of life. Because the knee joint is the most complex key of the human body, the anatomical structure of its ligaments and the individual form and size of femur, tibia, and patella lead to a gap between the satisfaction rate of TKA and total hip arthroplasty patients [18]. With the increase in age, the perioperative complications of total knee arthroplasty in elderly patients increase. Perioperative scientific preparation should be carried out to reduce the occurrence of such complications.

In this study, 15 patients with UKA and TKA were selected for efficacy comparison. In a retrospective study of patients receiving UKA and TKA, Casper et al. [19] found that compared with TKA, UKA had lower blood loss, more hemoglobin decreased, and more recessive blood loss in TKA. In this study, the perioperative indexes such as operation time, intraoperative bleeding, first time on the ground, and hospital stay in the UKA group were significantly lower than those in the UKA group. We could see that whether UKA or TKA, the postoperative VAS and HSS scores of the two groups were significantly improved compared with those before the operation, and the difference was statistically significant, indicating that the two knee arthroplasty methods could effectively improve the knee function, reduce pain, and achieve a satisfactory clinical effect. After comparing the ROM scores of patients before and after the operation, it was found that the ROM scores of patients in the two groups decreased significantly compared with those before the operation, and the ROM scores of patients treated with UKA were significantly better than those in the TKA group. It showed that both groups of patients could improve the postoperative knee range of motion, and UKA could improve the knee range of motion more than TKA. Because the anterior cruciate ligament plays an important role in knee kinematics, UKA retains the anterior cruciate ligament and retains more soft tissue and bone. This is an advantage over TKA [20, 21]. In the early postoperative complications, there was 1 patient with lower limb venous thrombosis in the UKA group, while 1 patient in the TKA group had delayed wound healing due to diabetes and 1 patient had a deep infection. Because the number of samples is general, there may be selective bias, the follow-up time is short, and there may be selection bias in the conclusion of postoperative complications. However, according to the reports of domestic and foreign literature, the early postoperative complications of UKA are less than those of the TKA group, and the postoperative recovery time of patients undergoing UKA is significantly earlier than that of TKA patients, which improves their quality of life and enables them to actively participate in society [22–24].

There are a few limitations to this study. Our results only included 30 patients, a small clinical sample. In addition, it is difficult to accurately assess the prognostic impact of TKA on KOA patients because of the lack of more follow-up records of patients after discharge and the lack of statistics on more indicators. This all requires us to further expand the clinical sample for the study.

## 5. Conclusion

Both UKA and TKA are very successful choices for the treatment of medial compartment knee osteoarthritis. However, according to the results of this study and many studies at home and abroad, UKA can better improve the knee range of motion and functional recovery and reduce the incidence of medical complications. Therefore, on the premise of strictly mastering the surgical indications, it is recommended to give priority to UKA lateral condylar replacement to improve the satisfaction of patients after knee arthroplasty.

## Figures and Tables

**Table 1:** Comparison of preoperative general data between the two groups.

	Gender		Age	BMI	Surgical side	
Group	Male	Female			Left	Right
UKA group (*n* = 15)	4	11	64.90 ± 8.35	25.39 ± 1.58	10	5
TKA group (*n* = 15)	4	11	69.80 ± 6.48	26.12 ± 1.46	6	9
*T*/*χ*^2^			−1.719	−0.269	2.143	
*P* value			0.108	0.802	0.143	

UKA, unicondylar knee arthroplasty; TKA, total knee arthroplasty; BMI, body mass index.

**Table 2:** Comparison of operation time, intraoperative blood loss, postoperative ground walking time, and length of hospital stay between the two groups (mean ± SD).

Group	Operation time	Intraoperative bleeding (ml)	Postoperative walking time (d)	Length of stay (d)
UKA group (*n* = 15)	55.65 ± 3.56	165.23 ± 18.06	9.16 ± 1.06	9.24 ± 1.86
TKA group (*n* = 15)	87.26 ± 5.29	216.34 ± 21.25	13.86 ± 1.57	14.56 ± 2.07
*T*	−7.480	−3.620	−4.297	−3.311
*P* value	0.002	0.022	0.013	0.03

**Table 3:** Comparison of HSS score and VAS score before and after operation (mean ± SD).

		UKA group (*n* = 15)	TKA group (*n* = 15)	*P* value
VAS	Preoperation	6.23 ± 1.28	6.58 ± 1.43	0.768
	5d after operation	1.54 ± 0.28	1.48 ± 0.33	0.830

*t*		6.200	6.028	
*P* value		0.003^*∗∗*^	0.004^*∗∗*^	
HSS	Preoperation	58.34 ± 2.62	58.29 ± 2.72	0.982
	5d after operation	88.27 ± 3.76	84.36 ± 2.68	0.216

*t*		11.295	11.825	
*P* value		≤0.001^*∗∗*^	≤0.001^*∗∗*^	

UKA, unicondylar knee arthroplasty; TKA, total knee arthroplasty; HSS, knee score scale; VAS, visual analogue pain scale. ^*∗∗*^*P* < 0.01, 5d after operation *vs.* preoperation.

**Table 4:** Comparison of knee ROM changes before and after operation (mean ± SD).

Group	Range of motion (ROM) of knee		*t*	*P* value
	Preoperation	5d after operation		
UKA group (*n* = 15)	109.43 ± 1.24	135.38 ± 1.67	−21.609	≤0.001^*∗∗*^
TKA group (*n* = 15)	109.58 ± 1.15	126.43 ± 2.06	−12.265	≤0.001^*∗∗*^
*P* value	−0.154	5.846		
	0.004^##^	0.580		

UKA, unicondylar knee arthroplasty; TKA, total knee arthroplasty; ^*∗∗*^*P* < 0.01, 5d after operation *vs.* preoperation. ^##^*P* < 0.01, UKA group *vs*. TKA group.

**Table 5:** Comparison of postoperative complications between the two groups.

Group	Number	Delayed wound healing	Deep infection	Lower limb venous thrombosis	Total (%)
UKA group	15	0	0	1	6.7
TKA group	15	1	1	0	13.3
*χ* ^2^	4.869				
*P* value	0.543				

## Data Availability

The data used to support the findings of this study are available from the corresponding author upon request.
